# Telomere-to-telomere assemblies of chromosome 10 reveal complex adaptive variation of 3-ketoacyl-CoA-synthases in *Populus trichocarpa* likely driven by Helitrons

**DOI:** 10.48130/forres-0026-0019

**Published:** 2026-06-08

**Authors:** David Kainer, Stanton Martin, Daniel Hopp, Sophie Mosher, Timothy J. Tschaplinski, P. Doug Hyatt, Madhavi Z. Martin, Jared M. LeBoldus, Kelsey L. Søndreli, Posy E. Busby, Mengjun Shu, Kerrie Barry, Jeremy Schmutz, Anna Furches, Nan Zhao, Daniel A. Jacobson, Jin-Gui Chen, Mirko Pavicic, Priya Ranjan, Wellington Muchero, Gerald A. Tuskan, Michael R. Garvin

**Affiliations:** 1 ARC Centre of Excellence for Plant Success in Nature and Agriculture, University of Queensland, Queensland 4072, Australia; 2 Biosciences Division, Oak Ridge National Laboratory, Oak Ridge, TN 37831, USA; 3 Oregon State University, Department of Botany and Plant Pathology, Corvallis, OR 97331, USA; 4 DOE Joint Genome Institute, Berkeley, CA 94720, USA; 5 The Bredesen Center for Interdisciplinary Research and Graduate Education, University of Tennessee, Knoxville, TN 37996-0184, USA; 6 School of Electrical Engineering, Southeast University, Nanjing 210096, China; 7 University of New Mexico, Albuquerque, NM 87131, USA

**Keywords:** Populus trichocarpa, Copy number variation, *KCS* genes, Helitrons, Long-read sequencing, Wax biosynthesis

## Abstract

The model woody plant *Populus trichocarpa* displays an atypical alkene-diverse wax cuticle likely driven by copy number variation (CNV) of *3-ketoacyl-CoA synthases* (*KCS*), which has been difficult to confirm with short-read assemblies. Long-read sequencing enables the development of telomere-to-telomere resources to detect cryptic variation, including CNVs, which are currently missed. Integrating this information can improve genomic prediction for breeding and provide insights into the evolutionary basis of important traits. Our analysis of 78 long-read haplotypes from chromosome 10 identified more than twice as many *KCS* genes as previously reported, and numerous intragenic non-synonymous substitutions. Random Forest predictive models highlighted the importance of *Potri.010G079500* in producing very long chain alkenes; however, its absence did not predict previously reported alkene-deficient phenotypes. Instead, alkene levels are best predicted by the combinations of *KCS* copies. Additionally, amino acid substitutions clustered around ligand and donor binding pockets, suggesting they contribute to differing wax cuticle composition. Finally, each *KCS* gene and copy was linked to a Helitron transposon. A phylogenetic analysis suggests Helitrons are the evolutionary mechanism for generating *KCS* tandem arrays. Long-read generated telomere-to-telomere assemblies of *P. trichocarpa* chromosome 10 revealed large-effect loci critical to genetic studies that are unattainable from short-reads. This new resource produced novel insights into genome structure and function, and a novel mechanism for generating tandem gene duplication. Our results highlight that, given current challenges in annotation and assembly, detailed and focused long-read sequences are key to interpreting complex genomic regions that contain tandem copy number variants.

## Introduction

The leaf cuticle, which is important for managing water use and colonization by bacteria and fungi^[1,2]^, contains an unusually large proportion (30%−40%) of very long-chain alkenes in *Populus trichocarpa* (black cottonwood). Recent studies have shown that wax levels and composition change during leaf development. Interestingly, in some *Populus trichocarpa* individuals, these alkenes are completely or nearly absent across all developmental stages^[3]^. This developmental variability could result from 1) phenotypic plasticity due to transcriptional regulation or 2) genomic/evolutionary adaptation. A previous transcriptomic analysis of *P. trichocarpa* genotypes displaying different developmental profiles of alkenes identified a cluster of *3-ketoacyl-CoA synthase* (*KCS*) genes that were significantly associated with the wax content of leaves, leading to the hypothesis that natural selection has driven the adaptive alkene phenotype. There is considerable interest in this locus as it has also been linked to several important traits, including bud flush, overall growth, drought tolerance, and disease resistance^[3−6]^.

The KCS enzyme is a substrate-specific protein within a multi-protein complex that iteratively elongates very long-chain fatty acids (VLCFA), subsequently leading to a pathway that forms long-chain alkenes^[7]^. Guo et al.^[7]^ identified *Potri.010G079500* as a likely candidate gene for the presence and absence of alkenes in the *P. trichocarpa* wax cuticle, along with four highly similar *KCS* genes (*Potri.010G079700*, *Potri.010G080000*, *Potri.010G080200*, and *Potri.010G080400*) arranged in a tandem array. Notably, *P. trichocarpa* has a locally high redundancy of *KCS* genes compared to other species, which may have arisen from a whole-genome duplication event or tandem duplications (local copy number increase). Accurate detection and verification of copy number variation (CNV) is challenging with short-read sequencing due to read mis-mappings^[8,9]^. Pan-genomic approaches, which survey genomic changes across an entire species, seek to integrate allelic variation that were excluded in single reference genomes. These previously undiscovered alleles hold the potential to disentangle the evolutionary history of genes^[4,10−12]^.

As part of our efforts to clarify the *KCS* locus in *P. trichocarpa*, PacBio sequencing was used to create telomere-to-telomere assemblies for 39 diverse genotypes (78 haplotypes) to resolve *KCS* copy number and structural variation at the individual haplotype level and relate these variants to cuticular alkene phenotypes. The selected genotypes vary in their cuticular alkene production, with some producing none and others that produce intermediate and high levels. Given this objective, our focus is on locus-level structural and functional variation, rather than population-genetic inference.

Here we report the precise map of the *KCS* gene variants on chromosome 10 and associate these genetic variants with alkene variation. Our analyses revealed more than twice as many *KCS* genes at this locus as those annotated on the 'Nisqually-1' v4.1 reference genome, including both CNVs and allelic variants. Integrating these CNVs and new allelic variants improved our ability to predict leaf alkene content. Placement of amino acid changes on AlphaFold-generated structures indicate that the allelic variants alter donor and ligand binding pocket affinities. Surprisingly, our efforts also revealed that Helitron transposons may be the evolutionary mechanism driving copy number increases. This work is evidence that a locus-centered approach, made possible by telomere-to-telomere assemblies of chromosome 10, can reveal evolutionary mechanisms and trait associations that complement broader genome-wide efforts.

## Methods

### Source material of *P. trichocarpa*

Plant material from 1,081 *Populus trichocarpa* genotypes, originally collected from wild populations in California, Oregon, Washington, and British Columbia were planted in a stool bed at the Oregon State University Research Farm in Corvallis, OR, USA^[4]^. During January 2014, dormant branch cuttings were collected and sent to the North Dakota State University's Agricultural experiment station research greenhouse complex in Fargo, ND, USA. For each genotype, branches were cut into 10 cuttings, measuring 10 cm in length, with at least one bud. Cuttings were soaked in distilled water for 48 h, planted in cone-tainers (Ray Leach SC10 Super Cone-tainers, Stuewe and Sons, Inc., Tangent, OR, USA) measuring 3.8 cm in diameter and 21 cm deep filled with growing medium (SunGro Professional Mix #8; SunGro Horticulture Ltd., Agawam, MA, USA) amended with 12 g of Nutricote slow release fertilizer (15-9-12) (N-P-K) (7.0% NH_3_-N, 8.0% NO_3_-N, 9.0% P_2_O_5_, 12.0% K_2_O, 1.0% Mg, 2.3% S, 0.02% B, 0.05% Cu, 0.45% Fe, 0.23% chelated Fe, 0.06% Mn, 0.02% Mo, and 0.05% Zn; Scotts Osmocote Plus; Scotts Company Ltd., Marysville, OH, USA). The cuttings were planted such that the uppermost bud remained above the surface of the growing medium. Plants were grown in a greenhouse with a temperature regime of 20 °C/16 °C (day/night) and an 18-h photoperiod supplemented with 600 W high-pressure sodium lamps. Slow-release fertilizer was added weekly with 15-30-15 (N-P-K) Jack's fertilizer (Jr. Peters Inc, Allentown, PA, USA) at 200 ppm for 2 months to promote root growth and subsequently fertilized with 20-20-20 (N-P-K) liquid fertilizer (Scotts Peters Professional; Scotts Company Ltd., Marysville, OH, USA), once a week. Plants were watered as needed.

### Sequence analysis

PacBio HiFi CCS reads were generated by the Joint Genome Institute and Hudson Alpha for 44 genotypes (88 haplotypes), with per-haplotype coverage ranging from 40.58× to 83.07× (mean 61.11×). Hi-C chromatin-capture libraries were also prepared, and assemblies were produced with hifiasm-HiC and polished with Racon. Chromosome 10 assemblies exhibited high structural continuity, with most haplotypes represented by one or two contigs and consistent chromosome lengths (~22 Mb). Whole-genome BUSCO analyses further indicated high completeness (96.6%–98.5%; Supplementary Table S1). Downstream analyses were restricted to 39 of the 44 genotypes due to the availability of complete and internally consistent associated phenotype datasets (Supplementary Table S1), from which the *KCS* locus was extracted (Supplementary Table S1). *KCS* genes were classified into *KCS1* and *KCS2* groups according to previously published phylogenetic frameworks^[3]^, which distinguish these classes based on sequence divergence and evolutionary history within the *KCS* gene family. Evolutionary analyses were restricted to KCS1 genes due to their pronounced copy number expansion and tandem duplication within the locus. In contrast, genotype-to-phenotype predictive models incorporated both *KCS1* and *KCS2* genes to evaluate whether structurally co-localized but previously underappreciated *KCS2* variants contribute to alkene phenotypes. This design enabled direct evaluation of whether prior functional interpretations based on gene class alone adequately capture locus-level phenotypic effects.

The first 30 nucleotides beginning from the ATG start codon are conserved in all five *KCS* genes (*Potri.010G079500*, *Potri.010G079700*, *Potri.010G080000*, *Potri.010G080200*, *Potri.010G080400*), and therefore, this sequence string was used to search for all possible *KCS* genes in each haplotype in the 350-kb region of chromosome 10. We then extracted 1,812 base pairs (the longest gene of the five), produced a multiple sequence alignment, removed the intron based on the intron/exon structure defined by the Nisqually-1 v4.1 reference, and translated the sequences. Pseudogenes were annotated as 'nonsense' versions of the gene, which included frame-shift mutations and large deletions. *KCS* variants described as 'shorter-length' represent shorter coding sequences relative to the Nisqually-1 reference gene model but retain intact start and stop codons and are therefore predicted to encode functional proteins. Variants containing premature stop codons, frameshifts, or large disruptive deletions were classified as nonsense/pseudogene variants and excluded from predictive modeling.

Once each haplotype was annotated, we scored each for presence/absence of all known *KCS* genes (0/1). We then combined haplotypes to generate diplotypes and summed the two scores to generate a 0/1/2 genotype per *KCS* gene. Neighbor-joining trees were generated in CLC Genomics Workbench and visualized with FigTree v1.4.4. Branch lengths represent genetic distance (substitutions per site), and node labels indicate bootstrap support values based on N bootstrap replicates. Helitron transposons were predicted with a BLAST search of the sequence Helitron-N4_PTr#RC/Helitron (position 10598323−10599590) taken from the repeatmask track of Nisqually-1 v4.1 against each haplotype. We included 15 bases upstream from Helitron-N4_PTr#RC/Helitron because it contained the standard CTAG palindrome at the 3' end of a Helitron.

### Metabolite phenotypes

Fully expanded leaves (leaf plastochron index 9 ± 1) were collected from black cottonwood (*P. trichocarpa*) genotypes in genome-wide association studies (GWAS) at Clatskanie, OR, USA, over three consecutive sunny days in July 2012, as described previously^[13]^. Briefly, aqueous ethanolic extracts (80%) were analyzed for metabolites by gas chromatography-mass spectrometry following trimethylsilylation. Both automated and manual data extraction were used to extract VLC alkene peaks, which were then normalized to the amount of internal standard (sorbitol) injected and mass of leaf extracted.

### Disease phenotypes

Disease severity, caused by *Melampsora* spp. and *Venturia populina* in *P. trichocarpa* was evaluated at the research plantation in Clatskanie, OR, USA. *Melampsora* spp. infections were measured in 2012 and 2014 using a scale, where 0 indicates no rust signs, 1–very light rust occurrence, 2–rust on most leaves in some small clusters, 3–rust dominant on most leaves, but no leaf necrosis, and 4–heavy rusting with necrotic spots on leaves. For *Venturia populina*, disease severity was assessed in 2013 with a scoring system: 0–no apparent *Venturia*, 1–a few leaves infected, 2–most leaves infected and a small number of petioles and terminal branch buds affected, and 3–the majority of the petioles and terminal buds infected, a common shepherd's crook appearance.

Three isolates of *Sphaerulina musiva* (MN-12, MN-14, MN-20), collected from three separate trees in near Garfield, MN, USA, were chosen for inoculation, based on preliminary virulence testing, and transferred from storage (−80 °C) onto K-V8 (180 ml of V8 juice [Campbell Soup Company, Camden, NJ, USA], 2 g of calcium carbonate, 20 g of agar, and 820 ml of deionized water) growth media, sealed with Parafilm (Structure Probe Inc., West Chester, PA, USA). Petri plates were placed on a light bench under full-spectrum fluorescent bulbs (Sylvania; Osram GmbH, Munich, Germany) at room temperature until sporulation was observed. Following sporulation, five 5-mm plugs were transferred onto another K-V8 plate and grown for 14 d under continuous light. There were a total of 200 plates for each isolate.

The experimental design was a randomized complete block design with four blocks. Plants were inoculated when they reached a minimum height of 30 cm (~54 d after planting). Plates containing isolates were unsealed, and ~1 ml of deionized water was added to the plate. Rubbing the media surface with an inoculation loop dislodged the spores, and the spore suspension was collected with a pipette. The spore suspensions were individually bulked from the three isolates at a concentration of 10^6^ spores ml^−1^ for each isolate. Plants were taken out of the greenhouse, and their heights were measured prior to inoculation, sprayed with an HVLP gravity-fed air spray gun (Central Pneumatic, Harbor Freight Tools) at 20 psi until the entire leaf and stem surface was wet (15 ml), and placed into a black plastic bag for 48 h. At 3 weeks post-inoculation, phenotypic responses were characterized by evaluating disease severity using a scale from 1 to 5. (1 = no disease/cankers, 2 = small necrotic lesions but resistant reaction taking place, 3 = small [larger than 2] with necrosis extending beyond resistant response, 4 = large necrotic lesions, 5 = stem girdling lesions occasionally with sporulation). Tests for differences of disease phenotypes were performed with a Student's t-test.

### Genotype-to-alkene models

Since VLC alkene production in this population appears to have a binary tendency (AM/AP), we fit an iterative random forest (iRF) to classify individuals as AM or AP based on their *KCS* genotypes. iRF iteratively applies a random forest (RF) model with a feature boosting procedure between each iteration and has been shown to outperform standard RF when ranking features according to importance scores^[14]^. This model was fitted using accessions with both genotype and metabolite data using the iRF v3.0.0 package in R with 1,000 trees and five iterations^[15]^. *KCS* genotypes were encoded as 0/1/2 for the iRF predictor matrix^[14,16]^. Furthermore, the AP individuals show a wide distribution of alkene abundances, so to determine which *KCS* genes were most associated with alkene variation, we then fit an iRF model for each of Z-9-pentacosene and Z-9-heptacosene abundances using the *KCS* genotypes for only the AP individuals. All iRF models were performed using the iRF v3.0.0 package in R with 1,000 trees and five iterations^[15]^. Importance scores for each model were then extracted from the iteration with the best fit determined by out-of-bag accuracy.

### Shapley analysis

Shapley analysis was applied to the outputs of each iRF model using the fastshap v0.1.1 package in R to explain the influence of the most important predictors on the dependent variable. Each shapley analysis used 500 Monte-Carlo repetitions. Visualizations were generated with the shapviz v0.9.3 R package^[17]^.

### Structural analyses

We used the AlphaFold structure U5G1R7 for *Potri.010G080200* (POPTR_010G080200 on AlphaFold). We aligned with the 2ix4 PDB file for *Arabidopsis thaliana* mitochondrial beta-ketoacyl ACP synthase hexanoic acid complex using the MatchMaker function in Chimera with a Smith-Waterman algorithm. Substrate and malonyl-CoA binding pockets as well as numbering for alpha-helices and beta-sheets were taken from Chen et al.^[5]^. Structure is shown as a dimer as in *Arabidopsis thaliana*. Predictions were performed in ChimeraX using AlphaFold^[18]^.

## Results

### Genotype selection and alkene phenotypes

**Figure 1:**
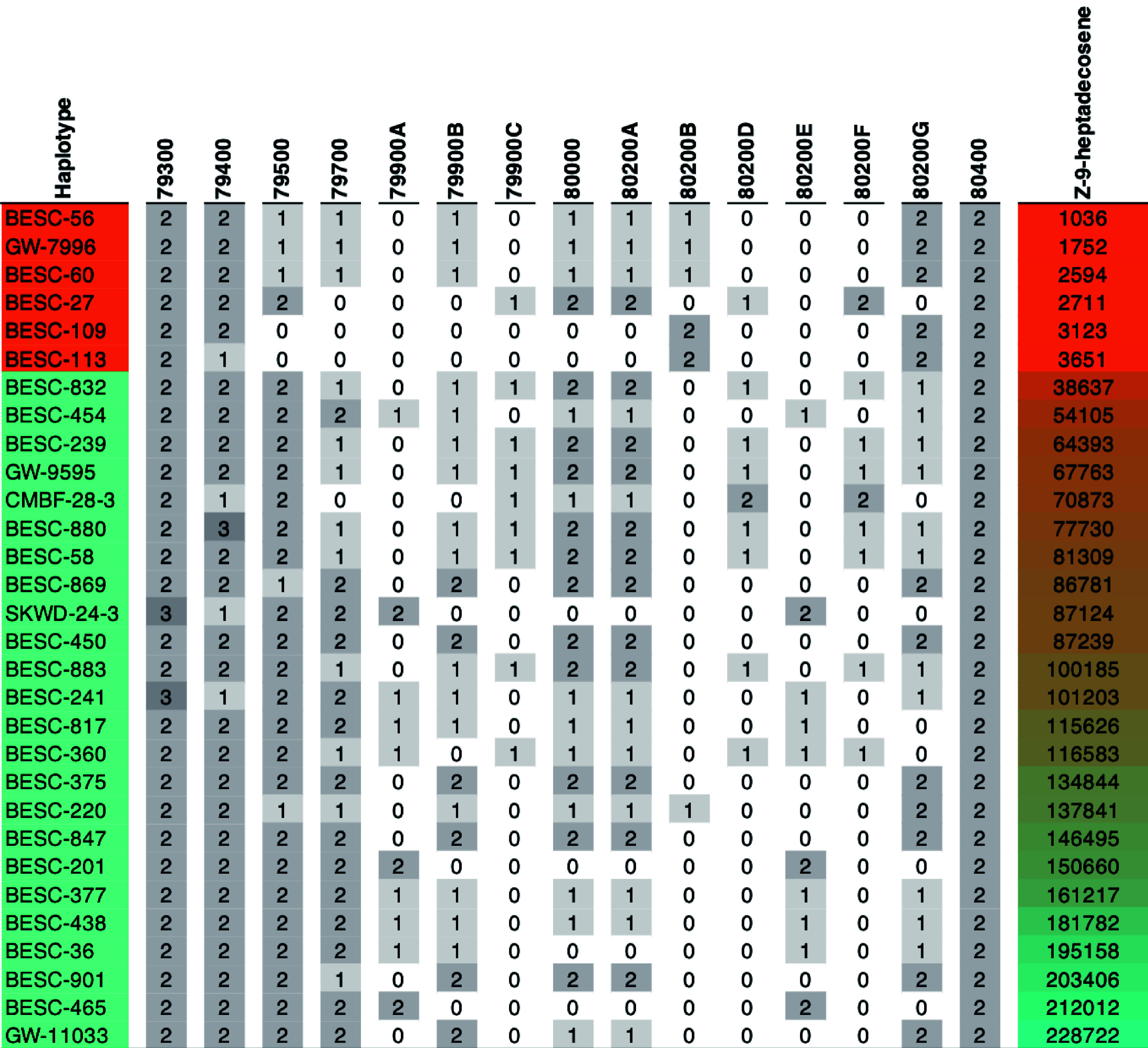
KCS gene copy counts for each genotype and their alkene levels colored by mean Z-9-heptadecosene amount. The first six genotypes are alkene minus (AM) accessions. The gray heatmap indicates copy numbers for genes listed as columns. A more detailed breakdown by differing amino acids is also provided in the Supplementary Table S2a.

**Figure 2:**
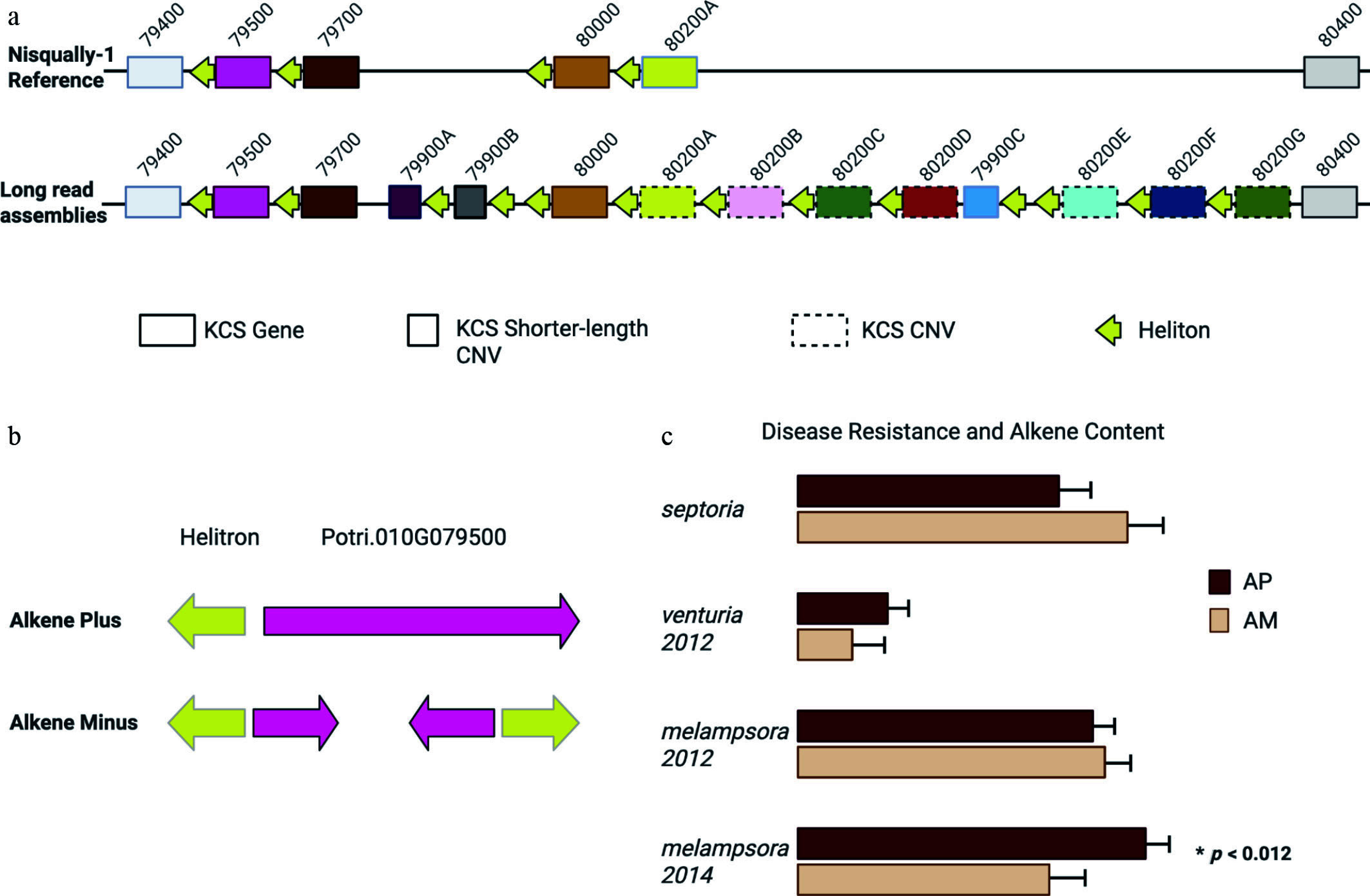
Comparison of our results to published reports of the KCS locus. (a) KCS genes in the Nisqually-1 reference and the new 78 haploid assemblies. Boxes with solid edges are genes (same position across genomes), boxes with broken edges are copy number variants (different positions across genomes), and short boxes represent shorter-length KCS variants with intact open reading frames. Nomenclature corresponds to Nisqually-1 reference. Yellow arrows indicate Helitrons with archetypal CTAG palindrome. Note, each gene is paired with a Helitron. (b) Gene model for *Potri.010G079500* in alkene-minus (AM) and alkene plus (AP) haplotypes. In AM individuals, the 5' end of *Potri.010G079500* was inverted and placed on the reverse strand, deleting portions of the coding sequence, confirming the prediction of Gonzales-Vigil et al. (c) Differences in disease severity in AM and AP phenotypes. Contrary to Gonzales-Vigil et al.^[3]^ we did not find differences in *S. musiva* (Septoria) severity but did find decreased severity of Melampsora in 2014 (*p* < 0.012) but not in 2012.

The long-read sequenced genotypes analyzed here were selected from a common garden population of 1,081 *P. trichocarpa* accessions and are representative of the genomic and phenotypic diversity, including variation in cuticular alkene abundance. Among the 39 genotypes included in downstream analyses, individuals span the full spectrum of alkene phenotypes, ranging from alkene-minus (AM) to intermediate and high in the alkene-plus (AP) class (Fig. 1). Alkene phenotypes were classified based on metabolomic measurements of very long-chain alkenes. Consistent with previous reports, AM individuals exhibit near absence of Z-9 alkenes, whereas AP individuals display a continuous distribution of alkene abundances (Supplementary Table S4). We additionally evaluated whether alkene phenotypes were associated with disease resistance. Contrary to earlier hypotheses, AM genotypes were not more susceptible to *Sphaerulina musiva*, although differences in Melampsora severity were observed in one sampling year (Fig. 2c).

### KCS locus structure and copy number variation

In total, 39 of the 44 genotypes were available for analysis, from which we extracted the 350-kb region that included the *KCS* locus. The *P. trichocarpa* v4.1 'Nisqually-1' reference genome contains seven *KCS* genes within this 350-kb region (10,400,000−10,785,000) on chromosome 10, comprising five *PotriKCS1* and two *PotriKCS2* genes. Here, we identified 14 different *PotriKCS1* genes (henceforth referred to as *KCS*) within this locus, seven of which are copy number expansions of *Potri.010G080200* (Fig. 2a, Supplementary Table S2a). The *KCS* sequences were classified as intragenic allelic variants if they carried variable sites but remained in the same genomic location. If the *KCS* gene differed both in sequence and genomic location, it was named a copy number expansion and assigned an alphabetic suffix. The size of the *KCS* locus varied greatly across haplotypes, ranging from 169 to 293 kb, which was significantly correlated with the total number of *KCS* genes and gene copies present (*p* < 1.5 × 10^−5^, Supplementary Table S2b). Among the 39 genotypes, five individuals carried a nonfunctional *Potri.010G079500* gene, where the 5' end of the gene had been partially copied and inserted into the opposite strand, deleting the 3' end of the gene (Fig. 2b). These five individuals were all alkene deficient.

### KCS gene features and annotation context

Comparison with prior genome annotations revealed several discrepancies. The original *Potri.010G079900* gene, reported in the Nisqually-1 v3.0 reference, lacked a stop codon and was subsequently removed in v4.1. Additionally, the v3.0 reference indicated that *Potri.010G080000* contained three exons, which was reduced to two exons in v4.1. Our findings confirm that the v3.0 annotation was correct, with this gene containing three exons (Supplementary Table S2a). Note, though, that among the 39 genotypes, several *KCS* genes, including those in Nisqually-1, have an indel that causes the loss of the first exon and therefore a non-functional protein.

### Helitron association with KCS genes

Previous reports identified a high number of Helitrons within the *KCS* locus^[3,5]^. Our analysis revealed that each *KCS* gene and gene copy is paired with a Helitron transposon, suggesting that *KCS* evolution and Helitron occurrence are co-occurrent (Fig. 2a). We initially identified 11 gene-Helitron pairs, but three Helitrons lacked a paired gene. A subsequent search for open reading frames identified three additional *Potri.010G079900* copy number variants, resulting in a total of 14 *KCS* genes*.*

**Figure 3:**
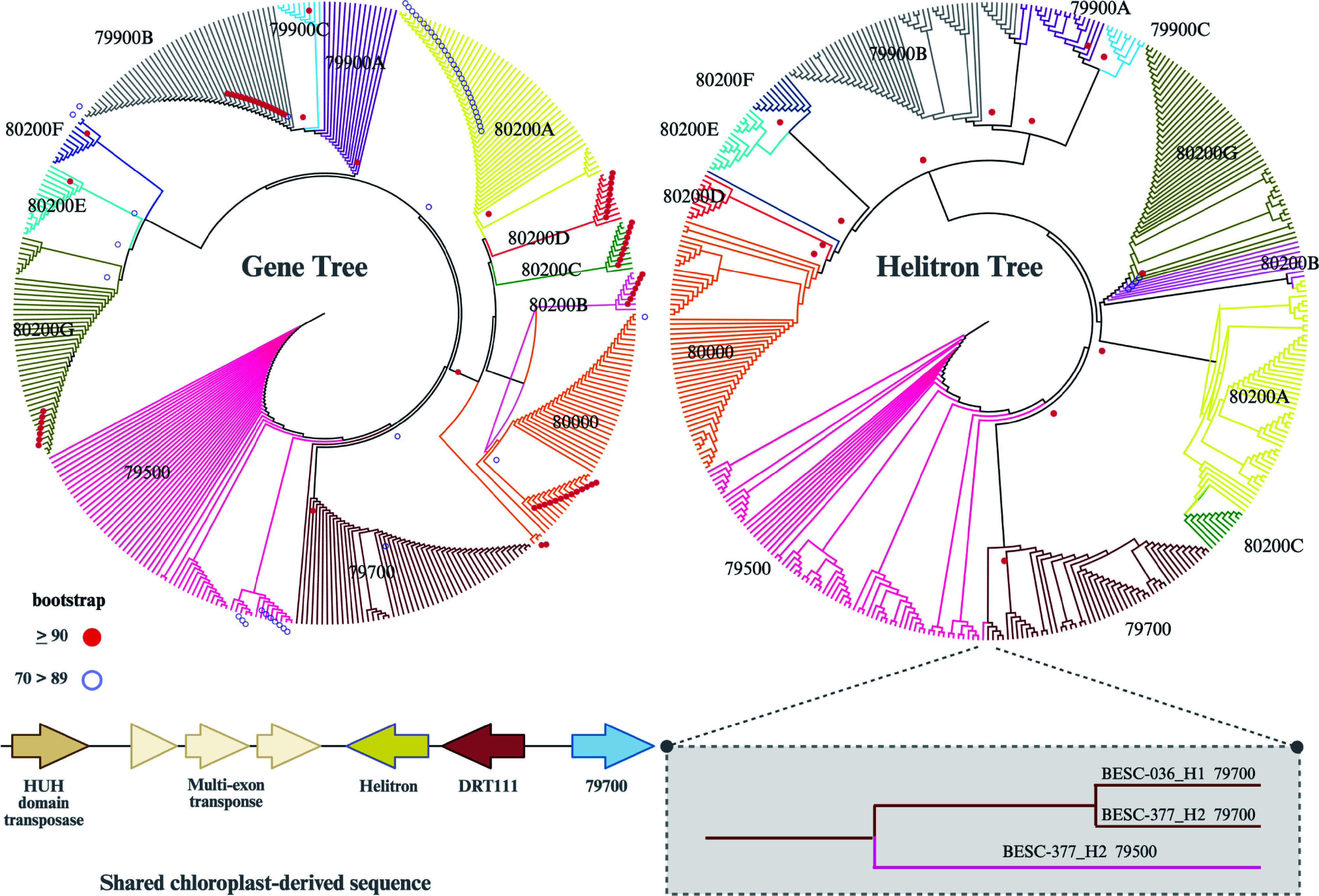
Neighbor-joining (NJ) trees based on exons of the genes and copy number variants (left) and their paired Helitrons (right). Colors correspond to those in Fig. 1a. Helitron sequences are mostly conserved, and the topology is congruent with the exon-based tree, with one major discrepancy (inset box). The coding sequences of *Potri.010G079500* and *Potri.010G079700* in 'BESC-377' vary, even though their Helitrons are nearly identical. The sequences shared between *Potri.010G079500* and *Potri.010G079700* are unique in that they both harbor a chloroplast-derived gene (DRT111) and two transposases. This DRT111-transposase sequence is found in all haplotypes with a full-length *Potri.010G079700* gene. In BESC-377_H2 it also appears upstream from *Potri.010G079500*, suggesting a shared evolutionary history. Bootstrap values for nodes greater than 90 and between 70 and 90 are shown.

To investigate potential co-evolution of Helitrons and the *KCS* genes, we generated neighbor-joining (NJ) trees for the genes and their paired Helitrons to determine if they were congruent (Fig. 3). The NJ trees, based on genes, and the tree based on the paired Helitrons, were highly congruent regarding topology, clustering by *KCS* gene, and copy number type. However, in one case, a Helitron from one gene was paired with a different *KCS* gene. The Helitron paired with the *Potri.010G079500* gene in haplotype BESC-377_H2 was nearly identical to the Helitron preceding *Potri.010G079700* in that same individual. Additionally, the upstream sequence contains several other unique features, including a copy of a chloroplast-derived *DRT111* gene, linked to adaptive traits from a previous genome-wide association study^[4]^. This unique upstream sequence also contains two transposases, one of which harbors an "HUH" domain that is indicative of Helitron transposition machinery^[19,20]^. This transposase is linked to the *Potri010G079700* gene across all haplotypes, *i.e.*, haplotypes lacking the *Potri010G079700* gene are either missing this transposase or have it partially deleted (Supplementary Table S3). Finally, contrary to previous reports, our results indicate that these alkene deficient genotypes are not more susceptible to *Sphaerulina musiva* compared to the full-length wildtype. They appear resistant to the leaf pathogen *Melampsora*, although our sample size is relatively small (Fig. 2c, Supplementary Table S4).

### Predictive models for alkene abundance using the KCS locus

**Figure 4:**
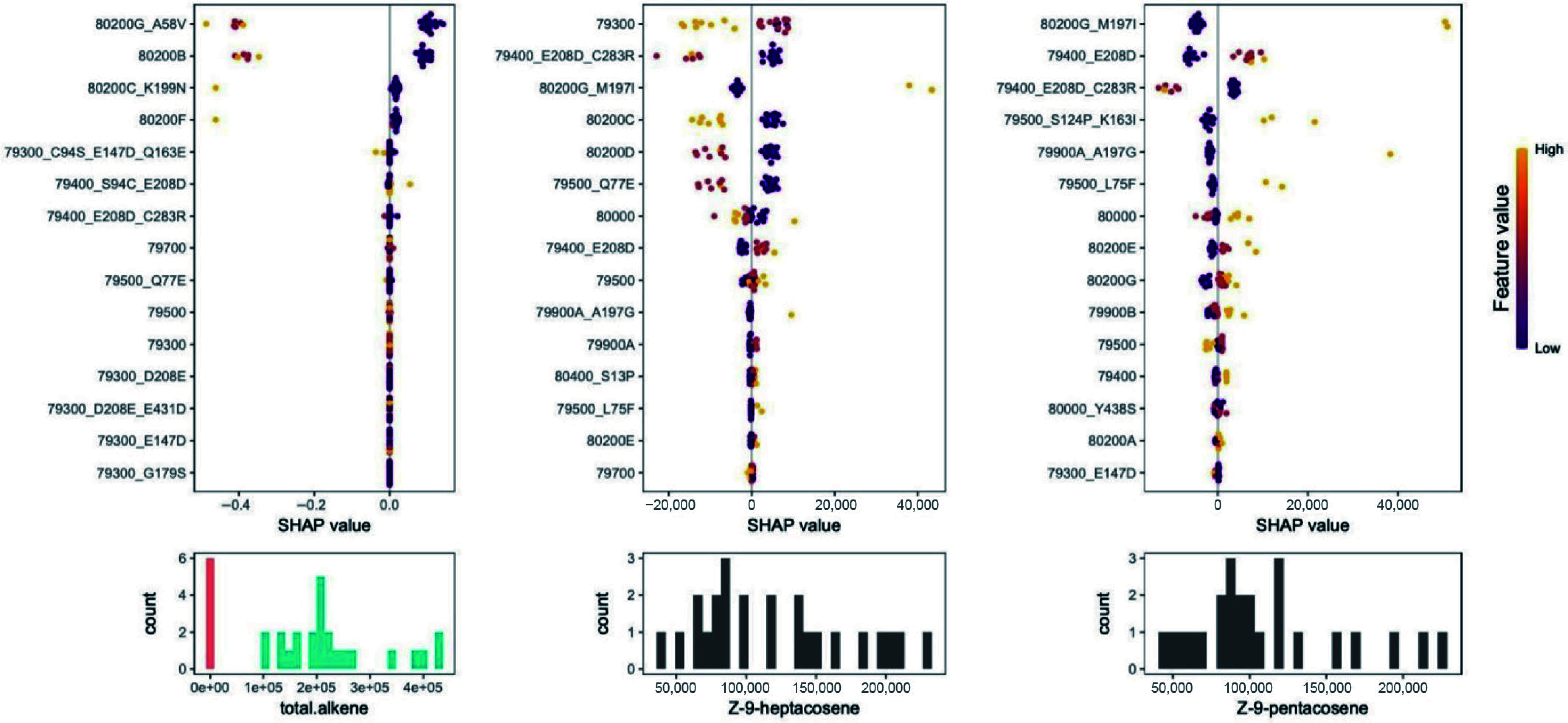
Explainable AI analysis of iterative Random Forest (iRF) models for different alkene phenotypes. The y-axis represents the SHAP value of each individual gene or copy number variant. Each filled circle represents an individual and the color represents the feature values, which is the number (dosage) of that gene or CNV for that individual. Purple indicates 0 alleles and yellow is the maximum number of alleles for a gene variant (usually two but can be one). Histograms below each SHAP plot depict the phenotype's distribution and SHAP values represent the tendency for that feature-allele combination to drive the phenotype prediction for an individual towards either low (left side) or high (right side) values. The model to predict alkene deficient (AM, red bars) vs alkene plus (AP, blue bars) phenotypes indicate 80200B and 80200G_A58V are the most accurate predictors for predicting AM or AP class (left plot), where non-zero dosage (1-red or 2-yellow) strongly drives individuals towards the AM class. The models that predict heptacosene (center) and pentacosene (right) identify a variant of the type 2 KCS protein Potri.010G079400 as important (E208D) for both, but addition of the C283R mutation decreases these alkenes. The presence of Potri.010G080000 has opposing effects on the two alkene types and different variants of the *Potri.010G079500* are important for predicting pentacosene levels but not heptacosene.

First, we predicted the binary trait of alkene-minus (AM) or alkene plus (AP) phenotypes by fitting an iterative Random Forest (iRF) classification model to determine which gene variants displayed the greatest importance based on all intragenic and copy number variation (Supplementary Tables S5, S6, Supplementary File). *Potri.010G080200* copy number variants *80200B* and *80200G_A58V* emerged as the most important predictors (Fig. 4, left). Contrary to expectations^[3]^, *Potri.010G079500* did not show significant predictive importance at the individual level. To clarify this observation, we fit iRF models to explain the variation in abundance of Z-9-pentacosene and Z-9-heptacosene, focusing only on individuals that produce the alkenes (i.e., AM individuals were excluded from this analysis). A wide variety of *KCS* genes and variants were found to be important in these models, including genes of both *KCS1* and *KCS2* classes (Fig. 4, center). However, only some genes were consistently important across both alkenes. That is, multiple variants of the gene *Potri.010G079500* were important for one alkene or the other but are clearly not the only drivers of alkene abundance variation at this locus. For example, having a double variant, 79400_E208D_C283R, in the *Potri.010G079400* gene, a more distantly related class 2 *KCS* gene^[3]^, corresponds to lower levels of both hepta- and pentacosene.

### Structural interpretation of KCS variants

**Figure 5:**
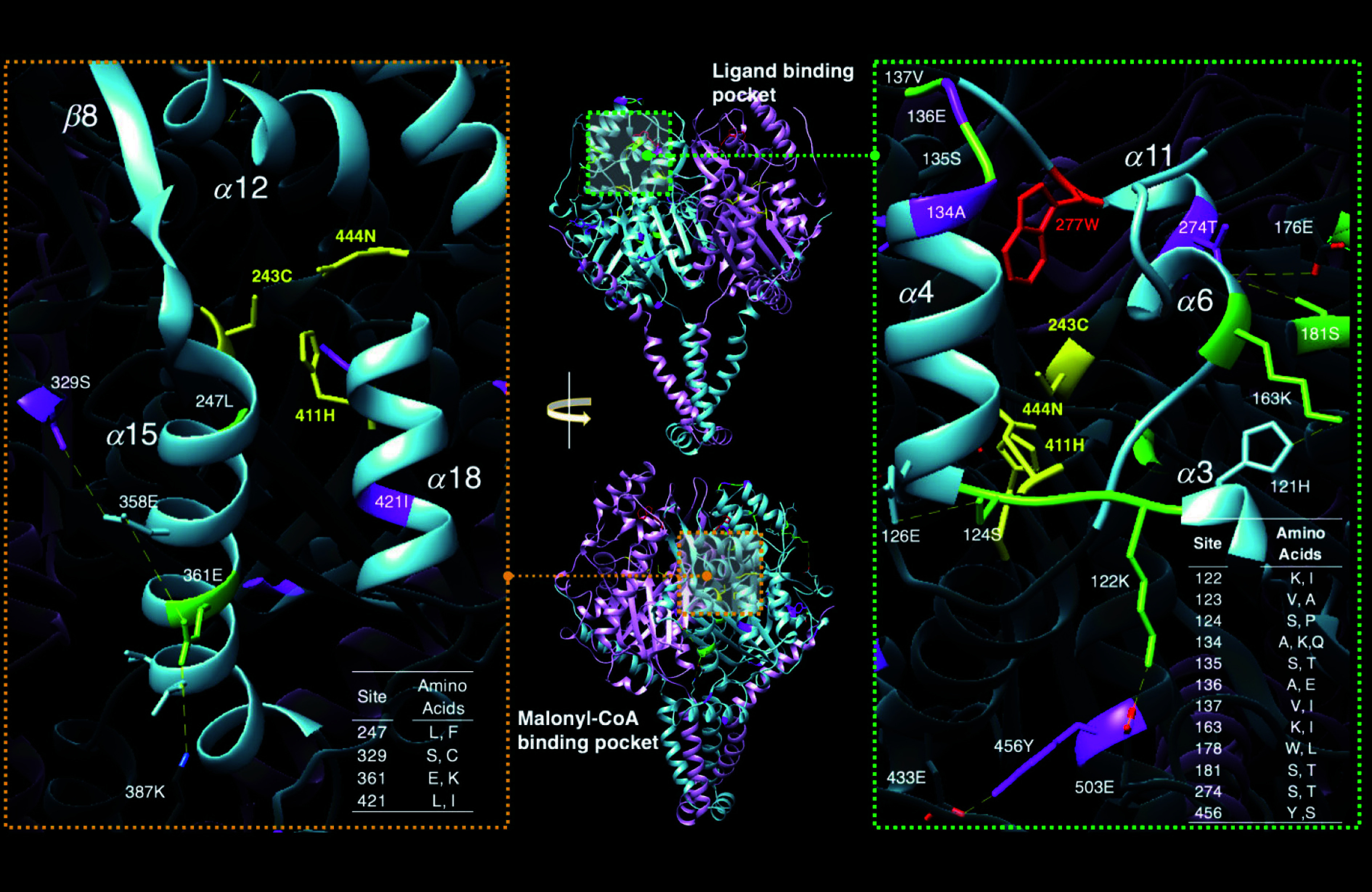
Amino acid substitutions overlaid on the AlphaFold predicted structure for *Potri.010G080200*. Previous work identified the binding pockets for the ligand (top, center panel) and the two-carbon donor malonyl-CoA (bottom, center panel). Substitutions among KCS variants in general (green residues in left and right panel) and those specific to the *Potri.010G080200* copies (pink residues left and right panels), cluster around the ligand binding pocket (magnified view with light blue coloring, right panel). The tryptophan at position 277 (as shown in red in the right panel) is necessary for function, active Cys-His-Asn triad (in yellow). Substitutions are also found in the malonyl-CoA binding pocket (magnified in the left panel). Changes are predicted to alter the hydrophobicity and shape of the region and likely affect ligand binding/activity (malonyl-CoA binding).

Interestingly, AlphaFold predictions suggest that the first 106 amino acids of the protein are disordered, making it unclear how amino acid changes here affect function. Supporting the hypothesis of adaptive selection of *KCS* genes, more than half (16/25) of the amino acid changes outside the disordered region cluster around the ligand binding pocket, with a few variants near the donor binding site for malonyl-CoA (Fig. 5, Supplementary Table S2). Many of the substitutions are predicted to alter hydrogen bonds, potentially impacting the active site directly. For example, the intragenic variant Y438S in Potri.010G080000 is predicted to alter a hydrogen bond with E433, repositioning the catalytic residue 444N (site 456 in Fig. 5) at the other end of the alpha helix on which it sits.

## Discussion

Our long-read, telomere-to-telomere analysis of *Populus trichocarpa* chromosome 10 underscores the central importance of accurate genome assemblies for linking genotype to phenotype. While our results corroborate aspects of earlier work on the *KCS* locus and very long-chain alkene production, they also challenge key assumptions and reveal additional layers of complexity.

Consistent with Gonzales-Vigil et al.^[3]^, we found that five of the six alkene-deficient individuals (AM) harbor structural variants that disrupt *Potri.010G079500* in at least one haplotype, likely reducing or eliminating transcription and explaining the lower average expression observed in their AM group. However, at the individual level, loss of *Potri.010G079500* does not fully account for the AM phenotype. Four AM individuals retain at least one intact copy of this gene, and one AM individual carries two functional copies. Re-examination of data from Gonzalez-Vigil et al.^[3]^ also supports this pattern: two of their AM individuals show measurable expression of *Potri.010G079500*, a finding that was obscured by reliance on the more limited v3.0 genome. Taken together, the evidence suggests that while *Potri.010G079500* contributes to alkene production, it is not solely determinative. Instead, our study identifies *Potri.010G080200B* and *Potri.010G080200G_A58V* as robust markers for predicting the AM phenotype, highlighting the importance of considering combinations of *KCS* copies rather than reliance on a single gene.

Notably, predictive performance differed between modeling approaches. Random forest regression models aimed at predicting quantitative alkene abundance showed limited accuracy, whereas classification models distinguishing alkene-minus (AM) and alkene-plus (AP) phenotypic groups performed substantially better, with an out-of-bag error rate of approximately 10%. This contrast suggests that variation at the *KCS* locus plays a dominant role in determining the binary AM/AP phenotype, while the precise abundance of alkenes likely reflects a more complex architecture involving additional loci, regulatory variation, and metabolic interactions. Achieving this level of resolution required haplotype-resolved long-read assemblies and extensive manual curation, highlighting the challenges future studies will face in developing robust genomic prediction models across larger populations.

### Helitrons as a mechanism for KCS gene expansion

**Figure 6:**
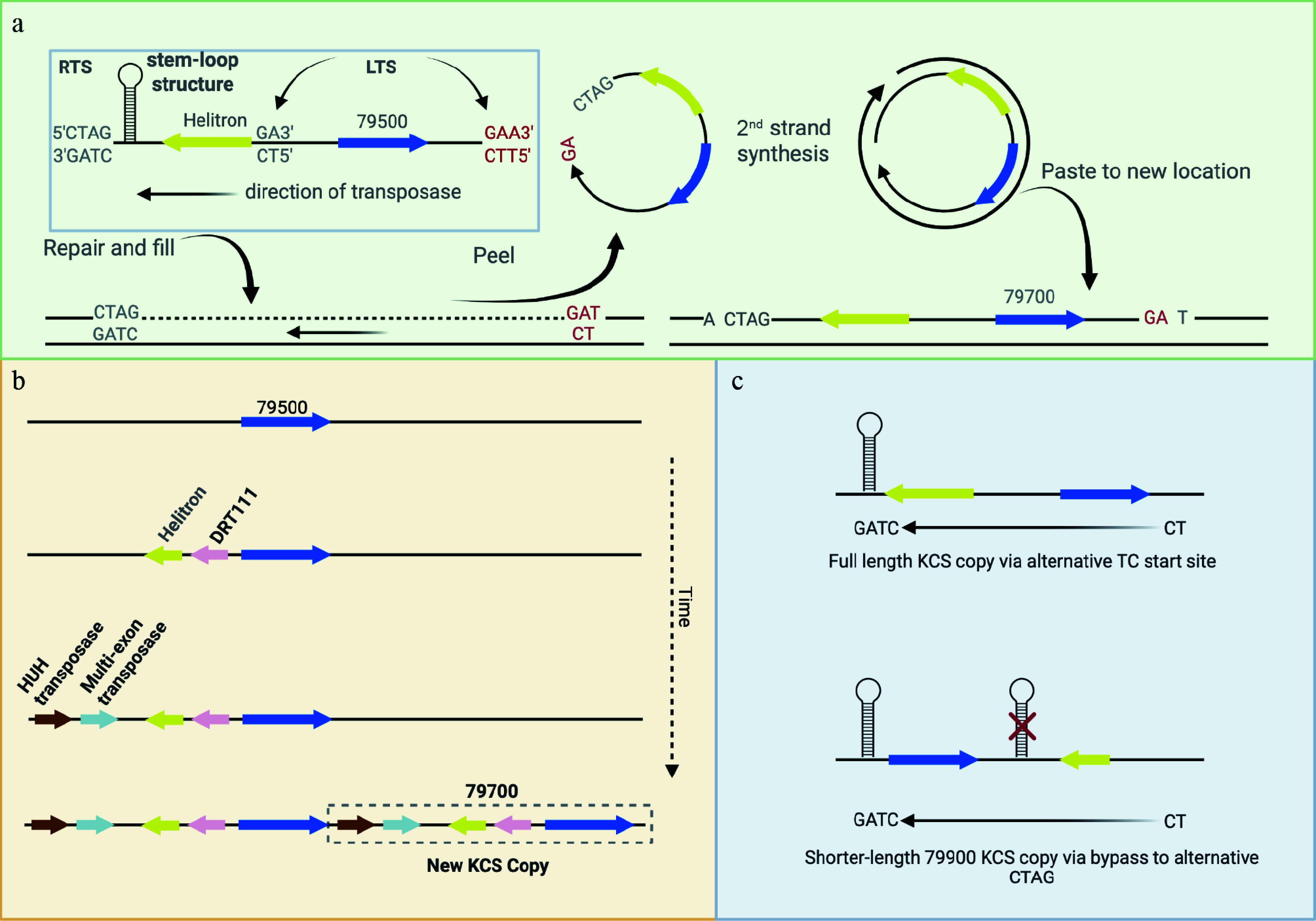
Model of possible mechanism of Helitron-driven copy number variation. (a) Helitrons have a unique peel-and-paste method of transposition in which a rolling circle is generated from a "TC" left terminal sequence (LTS) and a "CTAG" right terminal sequence (RTS) and inserted between an AT target nucleotide pair, with no duplications at the end (note KCS Helitrons are on the negative strand so LTS and RTS are reversed). The single strand from which it was derived is restored, retaining the original gene. Nearly all Helitrons harbor a 'T' upstream from the 'CTAG' site, and an 'ATC' just downstream from its KCS paired gene (Supplementary Tables S7, S8a, S8b), as would be expected if they were inserted as Helitrons (potential alternative LTS shown in red). (b) Hypothetical evolutionary model of copy number generation via Helitrons. Given the shared Helitron between *Potri.010G79500* and *Potri.010G079700* in BESC-377_H2 and the presence of that Helitron in all *Potri.010G79700* sequences, we propose that *Potri.010G079500* represents the origin of KCS copies. Once the transposase machinery and the DRT111 gene were inserted proximal to the Helitron in *Potri.010G79500* it was able to replicate itself and generate *Potri.010G79700*. (c) For the shorter-length KCS genes 79900A, 79900B and 79900C, the Helitron is downstream, rather than upstream and therefore a model consistent with Helitron replication would require a bypass of the original CTAG site.

A striking and previously unrecognized feature of the *KCS* locus is the presence of a Helitron transposon adjacent to every *KCS* gene. This genomic architecture suggests a mechanistic role for Helitrons in generating and maintaining *KCS* copy number variation (Fig. 6). Helitrons transpose through a peel-and-paste mechanism that, unlike other DNA transposons or retrotransposons, does not create flanking duplications at the insertion site^[21,22]^. This characteristic makes them particularly well-suited for producing additional functional gene copies with minimal risk of deleterious insertional effects. Their poor sequence conservation—limited largely to a 5′ CTAG palindrome and a 3′ hairpin—combined with their repetitive nature may explain why previous short-read based studies either misannotated or missed them.

Helitron-mediated gene duplication is well documented in maize^[23,24]^, and our observations strongly suggest a similar process at the *KCS* locus in *P. trichocarpa*. Most reported Helitrons are non-autonomous, requiring external transposase machinery. In plants, such machinery typically includes an HUH nuclease and a helicase-like domain. Although speculative, the transposase genes and chloroplast-derived DRT111-like repair genes adjacent to *Potri.010G79700* may encode the enzymatic components necessary for Helitron mobility in *P. trichocarpa*. Unfortunately, structural predictions for these mobile elements are not currently available through AlphaFold, limiting our ability to computationally assess their capacity for Helitron transposition.

### Limitations

This study required extensive manual curation and annotation, reflecting the current limitations of automated approaches in resolving complex genomic regions. Although algorithms can detect presence/absence and estimate copy number, identifying intragenic variation, structural nuances, and Helitron boundaries still relies heavily on expert manual review. For example, splice-site variation produced incorrect models of *Potri.010G80000* and led to the removal of *Potri.010G79900* in the v4.1 annotation, all of which required correction. While our revised gene models are strongly supported, definitive validation will require long-read transcriptome sequencing.

Similarly, our model in Fig. 6 is based on the conserved 'GAA' motif located immediately downstream of each *KCS* gene. The current genome annotation includes a Helitron at this location that does not extend into the coding region; however, long-read DNA and RNA sequencing will be needed to fully resolve the boundaries, activity, and mechanistic role of these Helitrons.

An additional limitation is sample size. Although our dataset comprised 39 individuals (30 with metabolomic data), the depth of haplotype-level information substantially expands current understanding of the genetic and evolutionary basis of alkene biosynthesis. Nonetheless, several important questions remain open. For example, are all *KCS* alleles expressed and translated, or does allele-specific expression influence phenotype? Do *KCS* proteins form specific hetero- or homodimers, as shown in *Arabidopsis*^[25,26]^, and what is the functional spectrum of the 105 possible dimer combinations in *P. trichocarpa*? How do these variants interact with other enzymes in the fatty acid synthesis pathway, and what is the full diversity of resulting lipid products? Are phenotypes such as disease resistance or growth effects directly attributable to wax cuticle composition or secondarily linked to upstream fatty acid variation (e.g., oleic acid levels)? These questions cannot be addressed using short-read shotgun approaches. Rather, they will require long-read transcriptomics, isoform-resolved expression studies, and proteomics. Finally, the rapid expansion and diversification of *KCS* copies create challenges for gene nomenclature. While intragenic variation follows conventional naming schemes, copy number variation will require community standards and experimental verification to ensure consistency.

## Conclusions

High-quality, telomere-to-telomere long-read assemblies fundamentally reshape our understanding of genome structure, function, and evolution in *Populus trichocarpa*. By resolving complex tandem arrays at the *KCS* locus, we discovered more than twice as many *KCS* genes as previously annotated, identified extensive intragenic variation, and uncovered Helitrons as a probable mechanism of tandem gene duplication. These findings challenge earlier assumptions about the genetic basis of alkene production and demonstrate that phenotypic variation is best explained by combinations of KCS copies rather than the presence or absence of a single gene.

Our results highlight the necessity of long-read approaches for accurate genomic prediction, trait dissection, and evolutionary inference in forest trees. As structural variation and transposable-element–mediated gene expansion continue to emerge as key drivers of adaptation, the resources and insights generated here provide a foundation for future research integrating long-read RNA sequencing, proteomics, and functional genomics. Ultimately, this work demonstrates that resolving complex genomic regions is essential for understanding and leveraging the genetic diversity that underlies ecologically and economically important traits in forest trees.

## SUPPLEMENTARY DATA

Supplementary data to this article can be found online.

## Data Availability

Data for this manuscript has been released at: https://doi.org/10.25983/2322563 or is available in the Supplementary Tables.
