# Supplementary material for: Telomere-to-telomere assemblies of chromosome 10 reveal complex adaptive variation of 3-ketoacyl-CoA-synthases in Populus trichocarpa likely driven by Helitrons
Source: For Res (Fayettev). 2026 Jun 8;6:e019. doi: 10.48130/forres-0026-0019 (PMC13253262; doi:10.48130/forres-0026-0019)

Supplementary Data

Importance scores for iterative Random Forest (iRF) models

Importance scores are reported for each iRF model described in the manuscript.

Model 1: Alkene Minus (AM) vs Alkene Plus (AP) Classification
This classifier model was trained using all accessions included in the study. Model performance was evaluated using out-of-bag (OOB) estimation, yielding an OOB error rate of approximately 10%, indicating strong discrimination between AM and AP phenotypic classes.

Model 2: Z-9-Heptacosene Regression (AP individuals only)
This regression model was trained exclusively on AP individuals to evaluate predictors of quantitative variation in Z-9-heptacosene abundance.

Model 3: Z-9-Pentacosene Regression (AP individuals only)
This regression model was trained exclusively on AP individuals to evaluate predictors of quantitative variation in Z-9-pentacosene abundance.

Importance scores reflect the relative contribution of predictors to model performance and are scaled according to the internal iRF feature importance metric.

Regression model results should be interpreted cautiously given the complexity of quantitative alkene abundance traits.

iRF Important scores using all AM and AP accessions

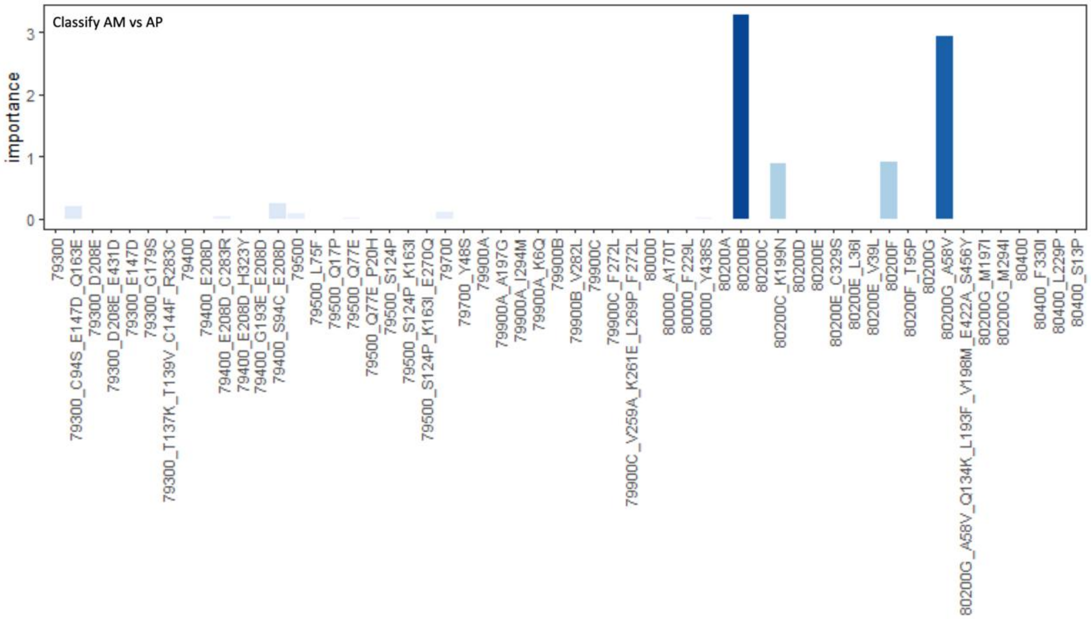

iRF Important scores using **only AP** accessions

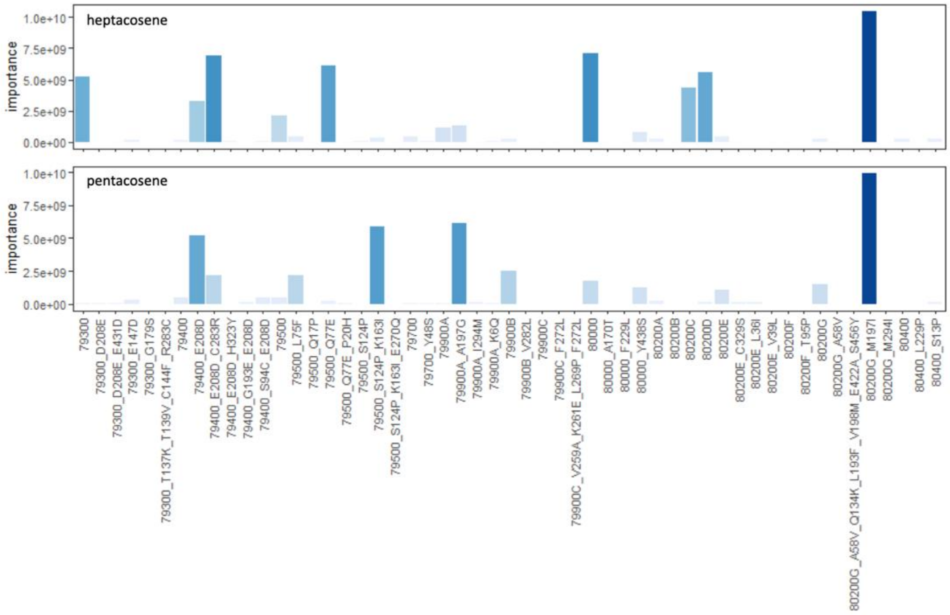

Supplement: Supplementary file 1 — Supplementary data to this article can be found online. [file forres-0026-0019-S1.zip › 10.48130_forres-0026-0019-Suppl-File1.pdf]
